# The evolution of antibiotic resistance in an incurable and ultimately fatal infection: A retrospective case study

**DOI:** 10.1093/emph/eoad012

**Published:** 2023-05-06

**Authors:** Robert J Woods, Camilo Barbosa, Laura Koepping, Juan A Raygoza, Michael Mwangi, Andrew F Read

**Affiliations:** Division of Infectious Diseases, Department of Internal Medicine, University of Michigan, Ann Arbor, MI, USA; Infectious Diseases Section, Veterans Affairs Ann Arbor Healthcare System, Ann Arbor, MI, USA; Division of Infectious Diseases, Department of Internal Medicine, University of Michigan, Ann Arbor, MI, USA; Division of Infectious Diseases, Department of Internal Medicine, University of Michigan, Ann Arbor, MI, USA; Department of Medicine, University of Toronto, Toronto, Ontario, Canada; Machine Learning Modeling Working Group, Synopsys, Mountain View, CA, USA; Department of Biology, Center for Infectious Disease Dynamics, Pennsylvania State University, University Park, PA, USA; Department of Entomology, Center for Infectious Disease Dynamics, Pennsylvania State University, University Park, PA, USA

**Keywords:** resistance, antibiotic, trajectories, *Enterobacter*, genetics, patient

## Abstract

**Background and objectives:**

The processes by which pathogens evolve within a host dictate the efficacy of treatment strategies designed to slow antibiotic resistance evolution and influence population-wide resistance levels. The aim of this study is to describe the underlying genetic and phenotypic changes leading to antibiotic resistance within a patient who died as resistance evolved to available antibiotics. We assess whether robust patterns of collateral sensitivity and response to combinations existed that might have been leveraged to improve therapy.

**Methodology:**

We used whole-genome sequencing of nine isolates taken from this patient over 279 days of a chronic infection with *Enterobacter hormaechei*, and systematically measured changes in resistance against five of the most relevant drugs considered for treatment.

**Results:**

The entirety of the genetic change is consistent with *de novo* mutations and plasmid loss events, without acquisition of foreign genetic material via horizontal gene transfer. The nine isolates fall into three genetically distinct lineages, with early evolutionary trajectories being supplanted by previously unobserved multi-step evolutionary trajectories. Importantly, although the population evolved resistance to all the antibiotics used to treat the infection, no single isolate was resistant to all antibiotics. Evidence of collateral sensitivity and response to combinations therapy revealed inconsistent patterns across this diversifying population.

**Conclusions:**

Translating antibiotic resistance management strategies from theoretical and laboratory data to clinical situations, such as this, will require managing diverse population with unpredictable resistance trajectories.

## INTRODUCTION

The effective treatment of bacterial infections has transformed modern medicine, but the ability of bacteria to evolve resistance to antibiotics threatens these gains [1]. For certain infections, the evolution of resistance within patients undergoing therapy can lead directly to treatment failure and worse patient outcomes [2–5]. Understanding how pathogens adapt during infections is crucial to identifying treatment strategies that impede resistance evolution and prevent possible transmission to others [6–8]. We previously described a patient with a chronic infection in which drug resistance evolution was fatal [5]. Here, we extend that case report by documenting genetic and phenotypic changes preceding that patient’s death and ask whether that knowledge, had it been available in real time, could have suggested additional treatment options.

**Figure 1.**
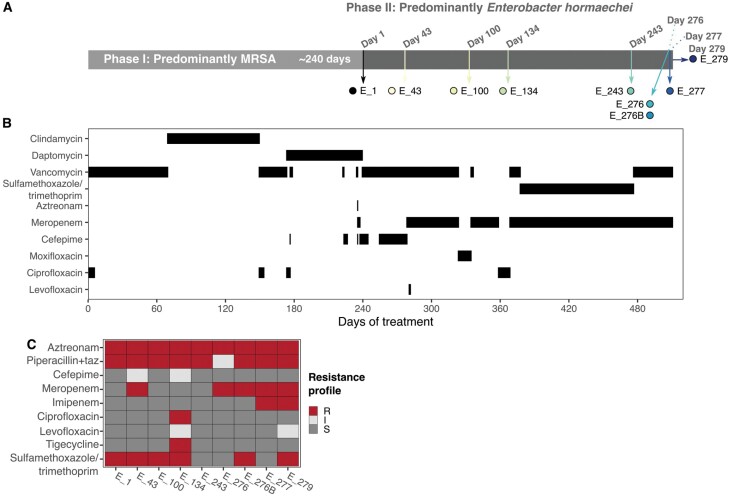
**Course of infection and *Enterobacter* isolation regime.** (a) The full course of infection consisted of two phases: The first infection phase (light grey) predominantly involved MRSA, lasting approximately 240 days. After this time *Enterobacter hormaechei* invaded the infection, initiating the second phase of infection (dark grey). Phase II lasted 279 days including seven hospital visits from which nine isolates were obtained (note the points and arrows have colours maintained throughout this manuscript). The day of isolation during the second phase is indicated at the top of the timeline. (b) Patent’s course of antibiotic treatment. (c) Resistance profile of the isolates as inferred from the CLSI breakpoints [12] for all isolates against nine different antibiotics, resistant (R), intermediate (I), and sensitive (S) isolates are shown in red, light grey, and dark grey, respectively

The infection developed in two phases (Fig. 1a): An initial period dominated by methicillin-resistant *Staphylococcus aureus* (MRSA) that lasted around 8 months and resulted in evolved resistance to clindamycin and daptomycin [5]. After 240 days, an *Enterobacter hormaechei* was cultured from the site of infection and became the only detectable pathogen in the subsequent 279 days. The *E. hormaechei* infection was treated with a variety of antibiotics (Fig. 1b; for review of clinical rationale behind choice and switching, see Ref. [5]). Resistance to various antibiotics arose, but the patient succumbed after resistance to meropenem evolved [5]. We asked four questions about resistance evolution in the *E. hormaechei* infection: (i) Did resistance emerge from the acquisition of *de novo* mutations or from genetic elements of other bacteria? (ii) Did the course of treatment select for a pan-resistant variant of *E. hormaechei* as previously described for other pathogens [9]? (iii) Was there a systematic change in resistance against particular antibiotics leading to substantial drops in resistance against others (i.e. collateral sensitivity [10],)? And (iv) were there drug combinations that could allow selection inversion approaches [11] be used? Various authors have suggested that collateral sensitivity could be exploited by judicious choice of antibiotics to prevent resistance evolution that leads to treatment failure [10] and that selection for resistance could be reversed (inverted) when mutations that increase resistant to one drug result in bacteria becoming more sensitive to a particular combination of antibiotics [11].

## METHODOLOGY

### Clinical isolates and antibiotics

We recovered nine isolates when the patient visited the hospital during the phase when *E. hormaechei* was detected. Labels, collection dates and source of isolation for each isolate are given in Table 1. We repeated MIC testing for the specific clones that underwent whole-genome sequencing and phenotypic testing (Fig. 1c), which may differ from those previously reported which were based on routine clinical phenotyping [5]. We systematically measured the phenotypic changes of the isolates using five of the most relevant drugs considered during treatment of the patient: cefepime, meropenem, ciprofloxacin, gentamicin and sulfamethoxazole/trimethoprim. For these selected drugs, we prepared stock solutions according to the manufacturer’s recommendations to be used in additional assays.

**Table 1.** Isolates, labels and sampling source

Isolate name	Day of isolation	Source of isolation
E_1	1	Blood
E_43	43	Blood
E_100	100	Blood
E_134	134	Blood
E_243	243	Wound
E_276	276	Blood
E_276B	276	Wound
E_277	277	Wound
E_279	279	Blood

### DNA extraction

We extracted genomic DNA using overnight cultures of each isolate in lysogeny broth (LB) and using the DNeasy Blood and Tissue kit (Qiagen) following the protocol for Genomic DNA Isolation from Gram-Negative Bacteria.

### Genomic analysis

Sequencing was performed at the University of Michigan Sequencing Core utilizing Illumina HiSeq and PacBio sequencing. We obtained PacBio long-read sequencing data for the first and last samples (E_1 and E_279). Illumina short-read sequences were obtained for all nine isolates using 100 bp paired-end reads and repeated for four isolates with 150 bp paired-end reads. We assembled the genomes of the first and last isolates as follows: assemblies were generated from the PacBio data using CANU, version 1.5 [13], circularized using circlator [14], and then polished with the obtained Illumina reads. The closed genomes for strains, E_1 and E_279, and the short-read Illumina data for all isolates were submitted to NCBI (accession numbers CP023569 and CP027111 for the assembled genomes). We identified genetic variants by mapping the short-read sequencing of each isolate back to the closed genome of the first isolate using bwa [15] and samtools [16]. Variants associated with antibiotic resistance and IS elements were identified using ResFinder [17] and ISfinder [18], respectively. We then evaluated the presence of foreign genetic elements via horizontal gene transfer (HGT) in two ways: Firstly, by aligning the assembled genome of the first and last isolate using bwa [15] we found that all regions had a read coverage >0*x*, thus indicating that no DNA sequences in the complete genome of the last isolate that is not present in the first isolate. Secondly, we mapped the short-read data of each isolate to the assembled genome of the first one using bwa [15]. Any unmapped reads were then extracted with samtools [16] and *de novo* assembled using spades [19]. We examined the obtained contigs and found for all isolates a ~5 kb phage phiX174 which is added as a control in the Illumina sequencing, there were typically fewer than nine contigs per sample, most of the contigs were less than 250 bp long and they predominantly consisted of homopolymers. This suggests that no foreign genetic material was transferred horizontally among the isolates.

### Changes in resistance

We determined the concentration inhibiting 90% of growth (IC_90_) using the broth microdilution method detailed in the CLSI standard M07 [12] for each of the *E. hormaechei* isolates. We added each of the isolates to microdilution plates containing 2-fold dilutions of each of the antibiotics in triplicate and incubated them at 36°C for 21 h in LB media. At this time, we measured optical density (OD_600_) using a plate reader (FLUOstar Omega from BMG Labtech). We then fitted Hill curves to the OD_600_ data to calculate the IC_90_ using the R platform [20] and the minpack.lm library [21].

### Checkerboard assay

To evaluate the susceptibility of the isolates to antibiotic we used checkerboard assays. We diluted increasing concentrations of any two drugs along the *X*- and *Y*-axis of a 96-well microtitre plate, leaving the last column as a blank (no added drug or bacteria). We evaluated all drug combinations of meropenem and the remaining four drugs (cefepime, gentamicin, ciprofloxacin and sulfamethoxazole/trimethoprim). After 21 h of incubation at 36°C, OD_600_ was measured in each well. We determined synergy by calculating the fractional inhibitory index using FIC = (MIC_A,combined_/MIC_A,alone_) + (MIC_B,combined_/MIC_B,alone_), where MICA and MICB correspond to the minimal inhibitory concentration of any drug A and drug B, respectively.

## RESULTS

Genomic analyses reveal that resistance evolution was not the result of HGT nor resulted in a pan-resistant pathogen.

**Figure 2.**
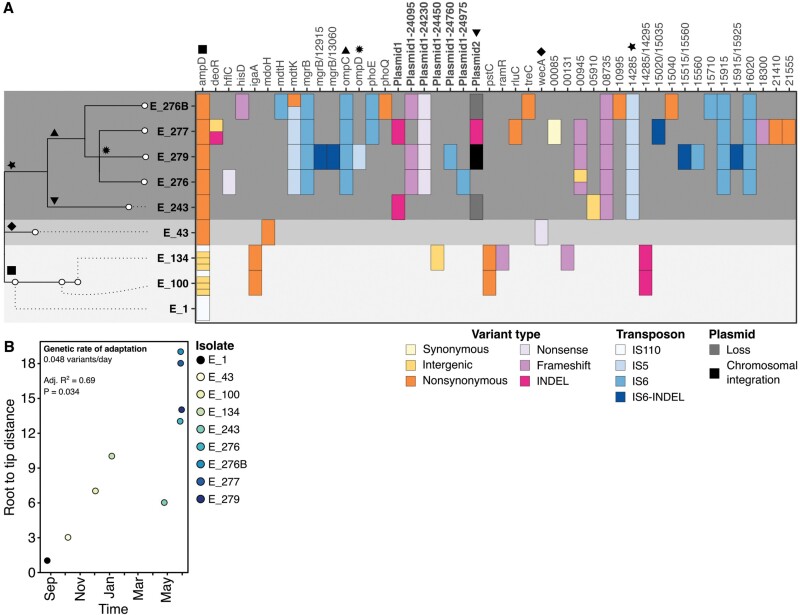
**Genomics of adaptation within a single patient**. (a) Maximum parsimony phylogeny of the nine *Enterobacter hormaechei* isolates taken from a single patient and projection of all the 58 variants in 40 genes of the nine isolates. Three major lineages were observed with no shared variants after day 243 (E_243) of infection with the *Enterobacter hormaechei* are highlighted in grey blocks. The lineage representative of the last 4 days, harbours more than half of the observed genetic variation. The different isolates are shown in different shades of yellow and blue, with the earliest isolate (E_1) shown in black. The variant type in the heatmap is indicated in different shades of orange and purple, transposon activity is indicated in different shades of blue and plasmid loss or integration is shown in dark grey and black squares. Genes in bold correspond to variants observed within plasmids. Symbols on the branches of the tree highlight genes potentially associated to changes in resistance against different antibiotics relevant for each lineage. b) Root to tip distance for each isolate over time

We found no evidence for the acquisition of foreign genetic elements by HGT. Instead, resistance evolved via the accumulation of point mutations, small INDELS and insertion sequence (IS) activity (Fig. 2a and Supplementary Table 1). Across the 9 examined isolates, 58 mutations were found in 39 chromosomal genes and 5 genes were encoded in one of the three plasmids found on the earliest isolate. A large fraction of all the identified variants were the result of transposon activity or plasmid loss: 15 insertions, deletions or rearrangements of three different IS elements (IS6-like, IS5-like and IS10-like), 5 large deletions (1.7–34 kb) adjacent to a copy of an IS6-like transposon, and the partial (~70% loss) or complete loss of two of the plasmids was identified. Of all mutations, we only found a single synonymous and seven intergenic variants while most of the remaining variants were strongly disruptive (Fig. 2a).

We generated a parsimony tree by hand using all variants identified, resulting in no homoplastic genetic changes showing three distinct lineages with no shared variants and an estimated rate of genetic adaptation of 0.048 variants/day (Fig. 2a and b). The two lineages represented by the early isolates were not subsequently detected. Overall, the final population was genetically diverse with more than half of the observed genetic variation being still polymorphic during the last 3 days of the infection.

Two identified mutations are associated with resistance against drugs that were not used during treatment, suggesting the potential evolution of collateral resistance. Isolate E_134 was resistant to tigecycline, an antibiotic that was not used (Figure 1b, c). This isolate had a frameshift variant in *ramR*, a TetR-like transcriptional regulator mediating the expression of *romA* and *ramA* (Fig. 2a). Loss-of-function mutations in *ramR* have been associated with multi-drug resistance in *Enterobacter*, *Klebsiella pneumoniae* and *Salmonella enterica* serovar *Typhimurium* [22–24]. Similarly, a variant was identified in the late isolate E_276B in *phoQ* (Fig. 2a), a gene commonly associated with resistance to colistin and some aminoglycosides [25]. Yet, neither of these antibiotics was given, and the isolates were not phenotypically resistant. Altogether, these data demonstrate that as this population evolved in response to the administered drugs, it also evolved heterogeneity that may impact success in future, changing environments.

Parallel evolution at the gene level was found for a single gene, *ampD*. All isolates had at least one variant in *ampD*, but there were three independent origins: an IS insertion 11 bp upstream of *ampD* (E_1 and descendant isolates), 9 bp insertion between bp 137 and 138 (E_43) and a single nucleotide polymorphism causing amino acid change T137K (E_243 and later isolates Fig. 2a). Mutations in *ampD*, a repressor of the AmpC β-lactamase, are well known to lead to increases in resistance against numerous β-lactams in distinct bacterial pathogens [26–29]. All isolates were indeed resistant to the commonly used β-lactams, aztreonam and piperacillin/tazobactam, but most retained sensitivity to carbapenems (Fig. 1c), a pattern consistent with AmpC overexpression [30].

Other identified variants have been associated with resistance to β-lactams. These include variants in *deoR*, *wecA*, and a large transposon-mediated deletion affecting *rcsC*, which are genes involved in cell wall synthesis, O-antigen production and cell division, and are commonly associated with virulence and β-lactam resistance [31–33]. The observed nonsense variant in *wecA* emerged early during the infectious process but was subsequently overtaken by different genotypes in the population, despite potentially conferring resistance against meropenem, the main treatment agent in this case (E_43; Fig. 2a and 1b). Similarly, the two variants in *deoR* observed late during the infection were not present in E_277, an isolate obtained 2 days later (Fig. 2a).

We identified mutations in genes known to confer resistance against carbapenems, a class of antibiotics that includes meropenem, the main treatment drug used in this case. The four latest isolates shared a transposon insertion within *ompC* (Fig. 2a), a gene coding for an outer membrane porin. Resistance against most carbapenems and some cephalosporins has been associated with decreased expression of this gene [34]. Additionally, some isolates had similar transposon events affecting *ompD* and *phoE*, a porin and a phosphoporin involved in β-lactam uptake, respectively [35–38].

Finally, vancomycin and sulfamethoxazole/trimethoprim treatments were not targeted at *E. hormaechei* but were included to reduce the risk of an MRSA re-emergence (Fig. 1b). Yet, these antibiotics, particularly the sulfamethoxazole/trimethoprim could still have affected the population structure. Indeed, there was a partial or complete loss of plasmids in most isolates within the later lineage. One of these plasmids (Plasmid 2) harbours genes associated with resistance against trimethoprim (*dfrA12*), aminoglycosides (*aadA2*), sulfonamides (*sul1*) and fluoroquinolones (*qnrS1*) [39]. The loss and integration of this plasmid coincided with changes in sensitivity against sulfamethoxazole/trimethoprim and ciprofloxacin (a fluoroquinolone), but not against gentamicin (an aminoglycoside) in the later isolates (Fig. 1c and 2a).

### Patterns of collateral sensitivity can be obscured by underlying genetic differences from distinct lineages

**Figure 3.**
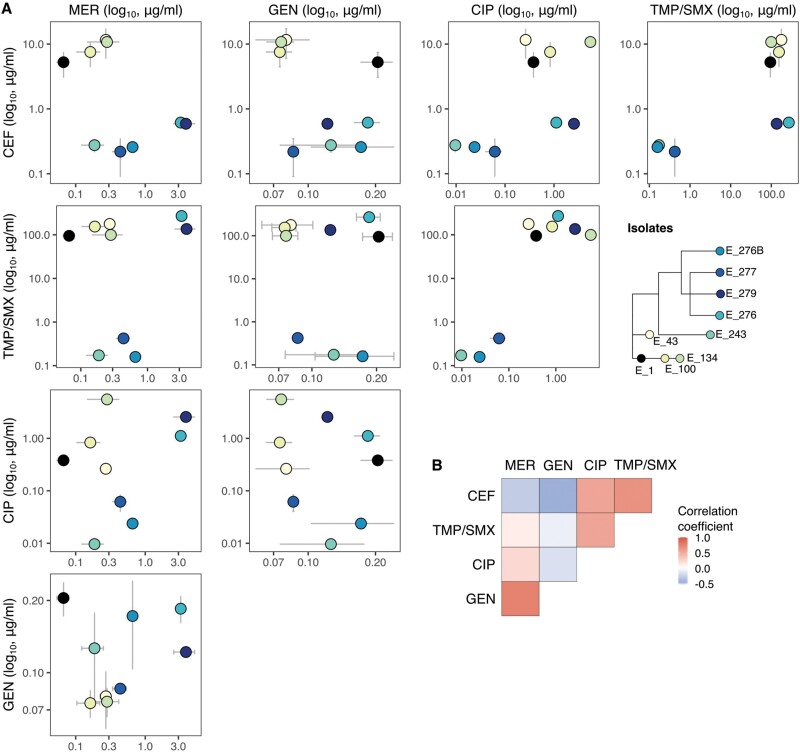
**Changes in resistance against five different antibiotics.** a) Concentration inhibiting 90% of growth for meropenem (MER), cefepime (CEF), gentamicin (GEN), ciprofloxacin (CIP) and sulfamethoxazole/trimethoprim (BAC) and the change in resistance in all isolates (arbitrary units) relative to the earliest isolate (E_1, in black). Points and error bars correspond to Mean IC_90_ ± SD of three technical replicates; isolate colours correspond to those shown in the tree. b) The Spearman’s rank correlation coefficient is shown in a heatmap for all possible associations between the five drugs (−0.5 < *ρ*_s_ < 0.78). After FDR correction for multiple testing no significant associations were found between the changes in resistance between the five drugs (*P* > 0.28)

Meropenem and cefepime showed a pattern of collateral resistance, with rare collateral sensitivity. The first four isolates, gathered when cefepime was used, evolved higher cefepime IC_90_, and at the same time gained collateral resistance to meropenem (Fig. 3a; top left panel). The later five isolates, obtained when treatment had predominantly shifted to meropenem, also showed a positive correlation between meropenem and cefepime IC_90_. However, comparing the early group to the late one, resistance against cefepime dropped several orders of magnitude, while sensitivity against meropenem increased slightly (Fig. 3a; top left panel). Thus, changes on this single branch that connect the early and later groups give an overall appearance of collateral sensitivity. This branch contains three genetic changes: a non-synonymous mutation in AmpD, T137K, a transposon insertion into CPT31_14285 a predicted glycosyltransferase and a frame shift in CPT31_08735, a TetR/AcrR family transcriptional regulator. Often, a TetR-like regulator represses the transcription of the divergently transcribed gene [40]. In this case, the divergently transcribed gene CPT31_08740 has homology to the multiple stress resistance protein BhsA. The meropenem-resistant isolates from the end of the infectious process also had higher levels of resistance against cefepime, yet they never reached the levels of resistance of the early group.

Similarly, the pairwise correlations across all nine isolates for all combinations of these five antibiotics revealed both positive and negative correlations (Fig. 3a and b). However, none of these associations were statistically significant (−0.5 < *ρ*_s_ < 0.78, *P* > 0.28; Fig. 3b), and careful inspection of the genetic changes reveals a more complex picture. Firstly, sensitivity against gentamicin remained below the initial levels of resistance of the earliest isolate, suggesting that increases in resistance against any other drug did not co-select for resistance against this drug. Secondly, the drastic drop in resistance against ciprofloxacin and sulfamethoxazole/trimethoprim coincides with the loss of plasmid 2 in some of the populations in the late lineage, which carries resistance against these drugs. Finally, none of the isolates showed phenotypic resistance against all the five antibiotics tested; isolate E_134 was the most resistant, having resistance to three of the five antibiotics tested (ciprofloxacin, sulfamethoxazole/trimethoprim and cefepime).

### No clear evidence for exploitable antibiotic combinations

**Figure 4.**
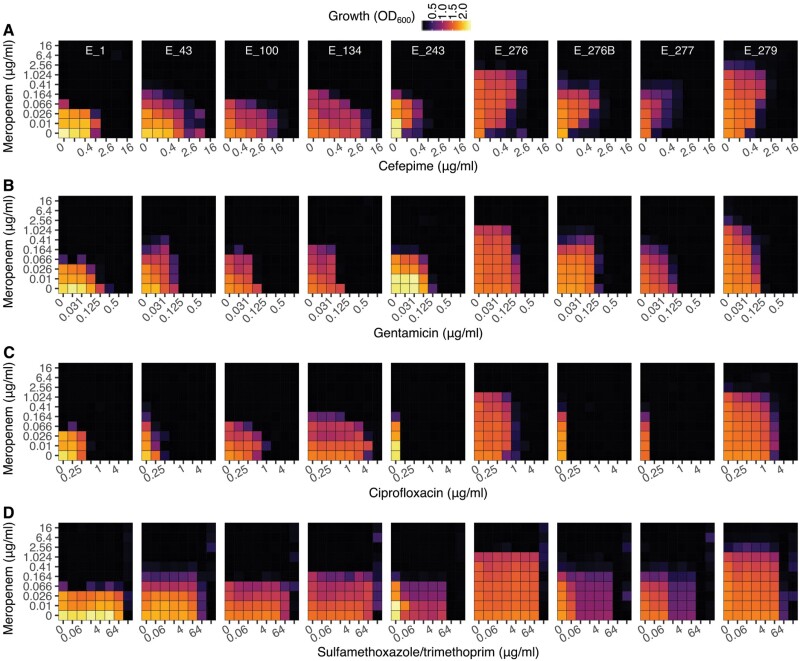
**Antibiotic interactions**. We measured growth in a grid of increasing concentrations of meropenem (*Y*-axis) and a) cefepime, b) gentamicin, c) ciprofloxacin and d) sulfamethoxazole/trimethoprim, (*X*-axis) using the checkerboard assay. We measured optical density after 21 h of incubation at 36°C. Areas of no growth are shown in black, with growth highlighted by the different shades of purple and yellow

We systematically evaluated combinations of each drug with meropenem using the checkerboard assay (Fig. 4). Most of the identified drug interactions for all isolates were additive, except for the first isolate when exposed to combinations of meropenem with cefepime (Fig. 4a), gentamicin (Fig.4b) and sulfamethoxazole/trimethoprim (Supplementary Table 2). Antagonism, which has a selection inversion potential [11], was only identified with meropenem and cefepime. In this combination, evolution amongst the early isolates reshaped the growth surface area by expanding growth into higher concentrations of both drugs, such that clones with increased resistance to meropenem also have increased resistance to cefepime at all concentrations (Isolates E_1, E_43, E_100 and E_134; Fig. 4a). However, in the later five isolates, a one-directional inhibition of antibiotic efficacy evolved, whereby small amounts of meropenem allow *E. hormaechei* to grow in much higher concentrations of cefepime. Amongst these later five isolates, the shape is similar, but there is a re-scaling along both the cefepime and meropenem axes. Thus, the opportunity for selection inversion exists between these two clades, but within each clade, there is a consistent pattern of collateral resistance. The patterns observed with the remaining three drugs similarly reveal that most of the phenotypic evolution is stretching or shrinking along one or the other axis, and, therefore, do not identify drug combinations that would allow for selection inversion (Fig. 4b–d). There are subtle shape changes, which correspond to the loss of plasmid 2 in some of the late isolates.

## DISCUSSION

We systematically described the evolutionary history of *E. hormaechei* culminating in multi-drug resistance within a single patient. We asked four questions. (1) Was resistance acquired by mutation or HGT? (2) Did a pan-resistant clone evolve? (3) Could antibiotic switching or (4) combination therapy have been clinically exploited to manage resistance evolution in this patient?

### Source of resistance

We found that resistance was the result of the accumulation of several mutational events—rather than through the horizontal acquisition of resistance genes. Resistance was a multi-step evolutionary process, with at least three distinct lineages accumulating multiple mutations. Consequently, the three lineages had important genetic and phenotypic differences. One lineage was observed early during the infection, showing steady increments in resistance against multiple antibiotics including cefepime and meropenem, which were the main treatment choices at the time. However, this early lineage was not seen again, apparently overtaken by a genetically distinct lineage that emerged between 134 and 243 days after *E. hormaechei* first appeared.

Apart from *ampD*, resistance was encoded in different genes, indicating that the population was able to explore the fitness landscape by taking multiple steps in multiple directions. Moreover, the bulk of the genetic changes were the result of plasmid loss, repeated transposon activity or mutations that were disruptive to gene function. This pattern is in line with previous findings in genomic epidemiology that have shown resistance frequently evolves *de novo* within individuals, without acquisition of resistance by means of HGT [6]. The observed patterns of gene disruption, gene loss and particularly the expansion of IS elements [41] bring into question whether the lineage present at the end could have competed outside of the patient.

The three lineages we detected differed from each other by a minimum of nine mutations. It, therefore, seems highly likely that it was their most recent common ancestor that infected the patient, and over the course of 279 days of infection gave rise to the resulting diverse lineages. However, in principle, we cannot rule out the possibility that the patient was infected separately two or more times by a closely related common ancestor. Moreover, our data demonstrate that the underlying structure of the pathogenic population was diverse. Our sampling allowed us to capture part of that diversity, but the use of clinically derived samples, which are limited to a single isolate per culture, give only a limited view of the evolutionary dynamics taking place. In the future, population genomics could represent an important tool to better dissect the total diversity within an evolving population.

### Pan-resistance

Ultimately, no single clone dominated the population, suggesting that mechanisms that can maintain diversity, such as niche differentiation, fitness costs of resistance and clonal interference played an important role during the evolutionary dynamics taking place within this patient. Moreover, the selected course of antibiotics did not lead to the emergence of a single clone with accumulated resistance to all the antibiotics used. Instead, resistance to different key therapeutic drugs emerged amongst different clones from the different lineages. This suggests that the use of multiple antibiotics may not consistently lead to the emergence of pan-resistant variants, as has been seen in *S. aureus*, where antibiotics are progressively selected for resistance in a stepwise manner within a single clone [42].

The potential evolution of pan-resistant bacteria has significant consequences for treatment: Pan-resistant clones can be impossible to treat. But an infection in which resistance to all antibiotics has been seen might still be treatable if no one clone has resistance to everything. For instance, ciprofloxacin resistance was acquired by one *Enterobacter* lineage but the lineage that was dominant when the patient died was apparently ciprofloxacin-sensitive (Fig. 1). For chronic infections, antibiotics against which resistance arose early in infections might be worth trying again, particularly when pan-resistance clones have not been detected.

### Antibiotic switching

When the evolution of resistance to one antibiotic is associated with susceptibility to another (collateral sensitivity), judicious switching of antibiotics during treatment could be used to mitigate resistance evolution [11]. Here, we identified a potential window of opportunity to do this during the transition between lineages. The late lineage had increased resistance to meropenem but decreased resistance to cefepime (Fig. 3a, top left panel). The transition between the early and late lineages represents an evolutionary trade-off that could potentially be exploited to push the population towards increased sensitivity against cefepime so that cefepime could be used therapeutically again. However, our analyses also identify the danger of such a strategy: while the between-clade pattern shows collateral sensitivities, the within-clade patterns support collateral resistance.

More generally, the potential consequences of changing treatment based on *in vitro* collateral sensitivity assays remain elusive. The bulk of *in vitro* studies evaluating collateral sensitivity indicate that evolutionary trade-offs are pervasive across many species, but its predictability depends on the specific genetic mechanism of resistance being selected [10, 43–45]. Furthermore, the effectiveness of strategies that leverage collateral sensitivity such as cycling regimes—alternating between antibiotics with known reciprocal collateral effects—may be reduced by negative epistatic interactions between resistance mechanisms against the different drugs [45]. Moreover, evidence of collateral sensitivity emerging in clinical infections is scarce, mainly because direct causation amongst drug use, genetic changes and changes in resistance phenotypes is difficult to demonstrate, even in longitudinal studies [46]. It is thus crucial to identify patterns of collateral sensitivity amongst clinical isolates while also evaluating different modes of implementation and side effects in meaningful and informative ways for clinical decision making.

### Combination therapy

Combination therapy may represent the best treatment strategy against infections when resistance emerges through *de novo* mutation, as observed in this patient. The effectiveness of combination therapy stems from the potential to control the population when resistance to several drugs requires independent mutations, as observed in other diseases [6]. In the treatment of this patient, therapy predominantly involved the use of more than one drug simultaneously [5] (Fig. 1b). However, this was motivated by the need to continue to suppress any remaining MRSA (Gram-positive bacteria) while targeting *E. hormaechei* (Gram negative) rather than for its potential to delay or evade antibiotic resistance evolution (Fig. 1a). Treatment typically consisted of vancomycin or sulfamethoxazole/trimethoprim to fight MRSA, and a β-lactam or fluoroquinolone to treat *E. hormaechei*. Interestingly, we found that despite sulfamethoxazole/trimethoprim treatment, isolates within the late *E. hormaechei* lineage lost a plasmid leading to reduced resistance to sulfamethoxazole/trimethoprim and ciprofloxacin (Figs. 1 and 2). This loss of sulfamethoxazole/trimethoprim resistance despite continued treatment points to incomplete understanding of drug penetration and selective pressure in this setting.

Could simultaneously using two or more drugs directly targeting *E. hormaechei* have controlled its resistance evolution during treatment? Different multi-drug strategies evaluated under laboratory conditions have been shown to reduce the selective advantage of resistance and reverse or delay the evolution of antibiotic resistance (reviewed in Ref. [11]). This selection inversion can only be achieved using multi-drug strategies, particularly when the chosen combinations interact suppressively (the combined effect is lower than that of one of the drugs) or synergistically with known evolved collateral sensitivity [11]. Our data suggest that for the first *E. hormaechei* isolate (E_1), most drug combinations, including meropenem showed weak synergy, with the exception of gentamicin that was additive (Fig. 4 and Supplementary Table 2). Importantly, this information was not available for clinical decision making at the time of treatment and so the possibility of resistance management by, for example, meropenem-cefepime combination therapy was not tested. However, these synergistic combinations were lost quickly and showed additive interactions instead in most of the isolates and combinations tested thereafter. Moreover, isolates from the late lineage showed that as resistance to meropenem evolved, increased sensitivity to cefepime and ciprofloxacin emerged and that when combined with meropenem, antagonistic suppression could be achieved (Fig. 4). So, selection inversion with combination therapy with meropenem and either cefepime or ciprofloxacin may have been possible in limited time windows, but does not represent a treatment strategy that was consistently available over the course of this infection.

## CONCLUSIONS AND IMPLICATIONS

Managing antibiotic resistance evolution in chronic infections remain problematic in clinical practice. Information on how pathogens evolve within single patients, such as the one presented here could lead to insights that may improve clinical decision making. We have described the main genetic and phenotypic changes occurring within a patient after a single run of the ‘evolutionary tape’ [47], and identified complex evolutionary trajectories, with evolution of collateral sensitivity and resistance, and idiosyncratic changes in drug-to-drug interactions over time. These results cannot tell us what would have happened had different treatment decisions been made along the way. Repeating this detailed mapping of treatment onto resistance evolution for many patients would strengthen causal inference, and perhaps justify controlled trials to test optimal treatment strategies. In the meantime, we note that had a genomic and phenotypic assessment of bacterial infections been available in real time while this patient was being treated, meropenem-cefepime combination therapy and/or reuse of ciprofloxacin may have been attempted. Whether those strategies would have limited resistance evolution and lead to better patient outcomes is unclear.

## Supplementary Material

eoad012_suppl_Supplementary_Tables
