# Supplementary material for: The evolution of antibiotic resistance in an incurable and ultimately fatal infection: A retrospective case study
Source: Evol Med Public Health. 2023 May 6;11(1):163–73. doi: 10.1093/emph/eoad012 (PMC10266578; doi:10.1093/emph/eoad012)
Supplement: eoad012_suppl_Supplementary_Tables [file eoad012_suppl_supplementary_tables.docx]

Supplementary Table 1: Genomic variants across all *E. hormaechei* isolates.

| **Isolate** | **Gene_name** | **Gene_id** | **Var_type** |
| --- | --- | --- | --- |
| E_1 | ampD | CPT31_04100 | IS110 |
| E_43 | wecA | CPT31_00845 | NONSEN |
| E_43 | ampD | CPT31_04100 | NONSYN |
| E_43 | mdoH | CPT31_08425 | NONSYN |
| E_100 | ampD | CPT31_04100 | IS110 |
| E_100 | ampD | CPT31_04100 | INTERGENIC |
| E_100 | ampD | CPT31_04100 | INTERGENIC |
| E_100 | ampD | CPT31_04100 | INTERGENIC |
| E_100 | z14285/z14295 | CPT31_14285/CPT31_14295 | INDEL |
| E_100 | igaA | CPT31_21550 | NONSYN |
| E_100 | pstC | CPT31_23350 | NONSYN |
| E_134 | ampD | CPT31_04100 | IS110 |
| E_134 | ampD | CPT31_04100 | INTERGENIC |
| E_134 | ampD | CPT31_04100 | INTERGENIC |
| E_134 | ampD | CPT31_04100 | INTERGENIC |
| E_134 | z14285/z14295 | CPT31_14285/CPT31_14295 | INDEL |
| E_134 | igaA | CPT31_21550 | NONSYN |
| E_134 | pstC | CPT31_23350 | NONSYN |
| E_134 | z00130 | CPT31_00130 | FRAMESHIFT |
| E_134 | ramR | CPT31_05845 | FRAMESHIFT |
| E_134 | Plasmid1-z24450 | CP023570-CPT31_24450 | INTERGENIC |
| E_243 | ampD | CPT31_04100 | NONSYN |
| E_243 | z08735 | CPT31_08735 | FRAMESHIFT |
| E_243 | z14285 | CPT31_14285 | IS5 |
| E_243 | Plasmid2 | CP023571 | PLASMID_LOSS |
| E_243 | Plasmid1 | CP023570 | INDEL |
| E_243 | z05910 | CPT31_05910 | INTERGENIC |
| E_276 | ampD | CPT31_04100 | NONSYN |
| E_276 | z08735 | CPT31_08735 | FRAMESHIFT |
| E_276 | z14285 | CPT31_14285 | IS5 |
| E_276 | mdtK | CPT31_09220 | IS5 |
| E_276 | mgrB | CPT31_12870 | IS6 |
| E_276 | ompC | CPT31_15015 | IS6 |
| E_276 | z15915 | CPT31_15915 | IS6 |
| E_276 | z16020 | CPT31_16020 | IS6 |
| E_276 | Plasmid1-z24230 | CP023570-CPT31_24230 | NONSEN |
| E_276 | Plasmid1-z24095 | CP023570-CPT31_24095 | FRAMESHIFT |
| E_276 | z00945 | CPT31_00945 | FRAMESHIFT |
| E_276 | z00945 | CPT31_00945 | INTERGENIC |
| E_276 | hflC | CPT31_02495 | NONSEN |
| E_276 | Plasmid1-z24975 | CP023570-CPT31_24975 | IS6 |
| E_276B | ampD | CPT31_04100 | NONSYN |
| E_276B | z08735 | CPT31_08735 | FRAMESHIFT |
| E_276B | z14285 | CPT31_14285 | IS5 |
| E_276B | Plasmid2 | CP023571 | PLASMID_LOSS |
| E_276B | mdtK | CPT31_09220 | IS5 |
| E_276B | mgrB | CPT31_12870 | IS6 |
| E_276B | ompC | CPT31_15015 | IS6 |
| E_276B | z15915 | CPT31_15915 | IS6 |
| E_276B | z16020 | CPT31_16020 | IS6 |
| E_276B | Plasmid1-z24230 | CP023570-CPT31_24230 | NONSEN |
| E_276B | Plasmid1-z24095 | CP023570-CPT31_24095 | FRAMESHIFT |
| E_276B | treC | CPT31_02890 | NONSYN |
| E_276B | phoE | CPT31_07945 | IS6 |
| E_276B | mdtH | CPT31_08495 | IS6 |
| E_276B | phoQ | CPT31_08825 | NONSYN |
| E_276B | mdtK | CPT31_09620 | NONSYN |
| E_276B | z10995 | CPT31_10995 | NONSYN |
| E_276B | z15040 | CPT31_15040 | NONSYN |
| E_276B | z15710 | CPT31_15710 | IS6 |
| E_276B | hisD | CPT31_17420 | FRAMESHIFT |
| E_277 | ampD | CPT31_04100 | NONSYN |
| E_277 | z08735 | CPT31_08735 | FRAMESHIFT |
| E_277 | z14285 | CPT31_14285 | IS5 |
| E_277 | mdtK | CPT31_09220 | IS5 |
| E_277 | mgrB | CPT31_12870 | IS6 |
| E_277 | ompC | CPT31_15015 | IS6 |
| E_277 | z15915 | CPT31_15915 | IS6 |
| E_277 | z16020 | CPT31_16020 | IS6 |
| E_277 | Plasmid1-z24230 | CP023570-CPT31_24230 | NONSEN |
| E_277 | z00945 | CPT31_00945 | FRAMESHIFT |
| E_277 | z00085 | CPT31_00085 | SYN |
| E_277 | deoR | CPT31_07370 | INDEL |
| E_277 | deoR | CPT31_07370 | INTERGENIC |
| E_277 | phoE | CPT31_07945 | IS6 |
| E_277 | z15020/z15035 | CPT31_15020/CPT31_15035 | IS6-INDEL |
| E_277 | z18300 | CPT31_18300 | FRAMESHIFT |
| E_277 | z21555 | CPT31_21555 | NONSYN |
| E_277 | Plasmid1 | CP023570 | INDEL |
| E_277 | Plasmid2 | CP023571 | INDEL |
| E_277 | z21410 | CPT31_21410 | NONSYN |
| E_277 | rluC | CPT31_08610 | NONSYN |
| E_279 | ampD | CPT31_04100 | NONSYN |
| E_279 | z08735 | CPT31_08735 | FRAMESHIFT |
| E_279 | z14285 | CPT31_14285 | IS5 |
| E_279 | mdtK | CPT31_09220 | IS5 |
| E_279 | mgrB | CPT31_12870 | IS6 |
| E_279 | ompC | CPT31_15015 | IS6 |
| E_279 | z15915 | CPT31_15915 | IS6 |
| E_279 | z16020 | CPT31_16020 | IS6 |
| E_279 | Plasmid1-z24230 | CP023570-CPT31_24230 | NONSEN |
| E_279 | Plasmid1-z24095 | CP023570-CPT31_24095 | FRAMESHIFT |
| E_279 | z00945 | CPT31_00945 | FRAMESHIFT |
| E_279 | Plasmid2 | CP023571 | PLASMID_INTEGRATION |
| E_279 | ompD | CPT31_11335 | IS5 |
| E_279 | z15560 | CPT31_15560 | IS6 |
| E_279 | mgrB/z13060 | CPT31_12870/CPT31_13060 | IS6-INDEL |
| E_279 | z15515/z15560 | CPT31_15515/CPT31_15560 | IS6-INDEL |
| E_279 | Plasmid1-z24760 | CP023570-CPT31_24760 | IS6 |
| E_279 | mgrB/z12915 | CPT31_12870/CPT31_12915 | IS6-INDEL |
| E_279 | z15915/z15925 | CPT31_15915/CPT31_15925 | IS6-INDEL |

**Supplementary Table 2. FICI scores for all isolates and four different combinations.**

**Synergistic interactions are shown in red, additive interactions in grey and antagonistic interactions in blue.**
